# Supplementary material for: A Likelihood Approach for Real-Time Calibration of Stochastic Compartmental Epidemic Models
Source: PLoS Comput Biol. 2017 Jan 17;13(1):e1005257. doi: 10.1371/journal.pcbi.1005257 (PMC5240920; doi:10.1371/journal.pcbi.1005257)
Supplement: S1 File — (TAR.GZ) [file pcbi.1005257.s014.tar.gz › HSPH_Online-SI-Revision/Additions/EnKF08/n100-extreme-SEITR_noisy/n100-extreme-SEITR_noisy_EnKF_table8.pdf]

1	R0	{1.38654, 9.85565}	{2.57508, 3.77769}	{{1.96361, 0.152057}, {3., 7.432}}	{85.7198, 63.8922}	inf	78.	3159.49	{318.92, 1600.93}	398.087	0.485949	newly	368.386	4716.47	{333., 4840.}	1191.54	0.771665	Reff	0.108483	0.493929	{0.0959058, 0.742077}	363.945	ar	92 379.	174 156.	{160 356., 189 699.}	88.523
2	R0	{2.36629, 12.2029}	{2.35629, 8.26768}	{{1.27745, 2.94524}, {3., 12.9207}}	{31.7592, 37.1993}	inf	5.	5138.58	{1318.89, 3276.24}	651.075	0.703859	newly	300.	4954.43	{1328., 5345.}	1555.39	0.936267	Reff	0.0235682	0.553272	{0.0776663, 1.24247}	2248.17	ar	99 022.	198 393.	{183 696., 233 811.}	100.353
3	R0	{2.32253, 12.836}	{2.55484, 3.87114}	{{1.85857, 2.49474}, {3., 4.92117}}	{25.5843, 69.8416}	inf	20.	3740.19	{332.901, 8688.34}	289.843	0.374759	newly	463.	6609.91	{326., 22 328.}	1340.97	0.656961	Reff	0.0245491	0.62763	{0.132027, 1.49395}	2456.86	ar	98 997.	186 288.	{166 566., 234 052.}	88.1753
4	R0	{1.79075, 11.2058}	{2.14395, 8.20442}	{{1.40134, 2.89122}, {3., 12.2443}}	{42.1357, 35.9597}	inf	31.	7801.17	{2591.63, 8980.14}	853.133	0.664457	newly	412.	7479.23	{1103., 6305.}	1724.3	0.813921	Reff	0.0574114	0.460592	{0.0500232, 1.00811}	708.274	ar	96 920.	183 467.	{164 476., 221 334.}	89.2978
5	R0	{2.26952, 9.62371}	{2.52208, 5.85226}	{{1.83435, 4.06}, {3., 7.54259}}	{26.8516, 39.1892}	inf	0.	2945.18	{149.5, 4164.26}	1483.44	0.883721	newly	78.	3858.35	{149., 5576.}	4847.17	1.1694	Reff	0.0257137	0.451785	{0.135891, 0.921898}	1661.89	ar	98 881.	181 427.	{167 185., 180 400.}	83.4802
6	R0	{1.57191, 6.89004}	{2.26502, 5.75291}	{{0.957879, 3.94912}, {3., 7.00509}}	{72.195, 18.4881}	inf	5.	2922.99	{1297.68, 3315.56}	5312.94	1.49783	newly	40.	3213.91	{1026., 2156.}	7934.78	1.61759	Reff	0.077951	0.611634	{0.180227, 0.957464}	689.373	ar	95 046.	188 923.	{177 459., 222 721.}	98.7702
7	R0	{1.99226, 17.2339}	{1.79533, 10.2521}	{{0.0560273, 3.40375}, {3., 15.8018}}	{69.5722, 40.5119}	inf	78.	6934.88	{984.372, 21 255.5}	172.789	0.338931	newly	982.	5332.93	{391., 13 106.}	468.317	0.51166	Reff	0.0456427	0.290448	{0.0136846, 0.706919}	566.862	ar	97 992.	173 402.	{160 195., 209 448.}	76.9556
8	R0	{2.63369, 15.1879}	{2.46531, 9.27278}	{{1.66323, 2.77794}, {3., 14.3554}}	{22.5272, 39.9729}	inf	5.	6666.98	{3826.16, 4835.56}	373.463	0.579153	newly	510.	7280.16	{1831., 11 528.}	1330.4	0.863077	Reff	0.0154861	0.571937	{0.144918, 1.62436}	3596.02	ar	99 451.	203 951.	{188 165., 262 969.}	105.077
9	R0	{2.53271, 9.90552}	{2.50703, 2.73265}	{{1.87238, 1.43637}, {3., 4.56629}}	{21.4079, 72.4129}	inf	2.	5691.89	{269.136, 3389.46}	3231.38	1.19464	newly	96.	12 858.3	{315., 42 388.}	13 294.6	1.57702	Reff	0.0167159	0.479745	{0.0618921, 0.708746}	2772.17	ar	99 355.	184 747.	{166 978., 247 947.}	85.9464
10	R0	{2.48791, 18.9003}	{2.58055, 13.3179}	{{1.95136, 4.11656}, {3., 17.7479}}	{20.9763, 29.5358}	inf	42.	8121.66	{1362.59, 7535.18}	138.121	0.324578	newly	1167.	4335.04	{770., 8340.}	283.422	0.463889	Reff	0.0212716	0.699415	{0.315602, 1.82397}	3188.02	ar	99 291.	178 254.	{156 999., 239 431.}	79.5269
11	R0	{2.84414, 18.4765}	{2.57558, 12.4227}	{{1.88408, 3.28056}, {3., 17.7534}}	{16.2378, 33.1225}	inf	13.	7694.09	{640.629, 14 152.4}	172.728	0.406451	newly	1003.	4819.75	{920., 8103.}	393.239	0.532364	Reff	0.0117463	0.723353	{0.0835508, 1.88435}	6058.13	ar	99 654.	184 251.	{157 341., 244 423.}	84.8909
12	R0	{2.29369, 14.622}	{2.54241, 7.73954}	{{0.584393, 4.47274}, {3., 13.082}}	{29.8239, 47.0692}	inf	14.	1309.39	{1073.95, 1730.47}	30.6547	0.148199	newly	542.	1237.9	{480., 1749.}	135.9	0.290324	Reff	0.0247948	0.447134	{0.0597472, 1.29599}	1706.28	ar	98 982.	177 970.	{167 785., 216 094.}	79.8003
13	R0	{2.73413, 16.7529}	{2.70621, 4.96302}	{{2.16059, 2.81195}, {3., 5.36919}}	{13.6626, 70.8837}	inf	10.	6312.35	{2338.28, 5577.78}	219.224	0.390652	newly	706.	7188.92	{1873., 13 497.}	923.184	0.82475	Reff	0.0138347	0.601591	{0.233758, 1.26753}	4250.21	ar	99 543.	210 856.	{191 174., 279 935.}	111.825
14	R0	{1.79554, 16.5943}	{2.52517, 7.05785}	{{1.89575, 3.04382}, {3., 18.1667}}	{40.6358, 60.5214}	inf	185.	4693.1	{2225.22, 9857.77}	74.3222	0.253023	newly	1313.	6599.98	{1473., 8844.}	408.005	0.484899	Reff	0.0662734	0.78041	{0.234077, 1.69955}	1078.03	ar	96 821.	208 330.	{187 107., 258 335.}	115.171
15	R0	{1.91429, 13.7771}	{1.92903, 3.97954}	{{0.382689, 1.24927}, {3., 4.6175}}	{66.1552, 71.1148}	inf	37.	2904.94	{23.6175, 3556.78}	136.692	0.507908	newly	621.	2869.65	{22., 4195.}	395.641	0.730997	Reff	0.0501544	0.301995	{0.0643149, 0.491957}	506.094	ar	97 541.	169 261.	{161 621., 174 790.}	73.5279
16	R0	{1.42094, 10.5871}	{2.46847, 9.07847}	{{1.18636, 3.41413}, {3., 16.9878}}	{80.9862, 49.3032}	inf	126.	10 902.3	{921.812, 10 767.4}	818.119	0.791504	newly	644.	7419.09	{889., 12 846.}	1057.51	0.862992	Reff	0.103388	0.427449	{0.160511, 1.09346}	319.886	ar	93 044.	182 054.	{161 773., 202 587.}	95.664
17	R0	{1.45515, 6.16466}	{2.56857, 4.04438}	{{1.87255, 3.42979}, {3., 4.42395}}	{76.5157, 34.3941}	inf	5.	3026.67	{149.026, 1225.68}	5953.4	1.24353	newly	41.	5457.82	{141., 2163.}	13 211.8	1.38747	Reff	0.0982954	0.520763	{0.219738, 0.823915}	436.542	ar	93 254.	170 922.	{158 110., 178 239.}	83.2862
18	R0	{2.85669, 13.2416}	{2.10651, 8.00289}	{{0.876189, 3.69632}, {3., 11.4484}}	{31.9795, 39.5625}	inf	1.	4308.23	{298.313, 4424.72}	483.618	0.543255	newly	314.	4268.65	{166., 5050.}	1281.16	0.757212	Reff	0.0106269	0.687674	{0.132425, 0.910375}	6371.08	ar	99 644.	173 372.	{158 423., 179 481.}	73.9911
19	R0	{2.56417, 17.9558}	{2.36626, 3.16863}	{{1.22442, 1.24471}, {3., 4.12263}}	{29.2939, 82.3532}	inf	35.	2086.13	{588.178, 3366.47}	93.3413	0.597956	newly	1136.	3877.86	{726., 10 594.}	273.893	0.341853	Reff	0.01818	0.552558	{0.0189678, 1.21048}	2945.23	ar	99 389.	154 077.	{144 048., 179 291.}	55.0239
20	R0	{2.27517, 11.1864}	{2.02921, 3.27911}	{{1.01803, 0.978763}, {3., 5.79499}}	{43.3061, 71.3498}	inf	9.	7664.49	{2777.62, 17 864.}	1566.19	0.943644	newly	219.	12 129.9	{2193., 56 003.}	5438.79	1.39576	Reff	0.0255502	0.538385	{0.204041, 0.834613}	2007.17	ar	98 894.	194 516.	{174 819., 251 258.}	96.6917
21	R0	{2.35735, 10.4735}	{2.51764, 4.032}	{{1.79354, 3.28824}, {3., 4.65343}}	{25.9142, 61.5028}	inf	3.	5489.15	{2477.43, 6563.77}	1737.64	1.04073	newly	156.	7489.44	{1966., 11 669.}	4702.26	1.35936	Reff	0.0230549	0.449457	{0.0259342, 1.41084}	1851.64	ar	99 036.	184 123.	{167 239., 227 863.}	85.9155
22	R0	{1.79551, 11.9179}	{2.74323, 3.04481}	{{1.9729, 2.24335}, {3., 4.09899}}	{52.7825, 75.6225}	inf	31.	2526.23	{711.446, 3484.31}	199.319	0.453681	newly	400.	3662.93	{636., 5469.}	821.008	0.822689	Reff	0.0555172	0.428464	{0.056716, 0.753034}	674.618	ar	97 033.	187 181.	{179 763., 197 431.}	92.9045
23	R0	{1.23545, 6.31687}	{2.66749, 10.0398}	{{2.19952, 3.85652}, {3., 12.8896}}	{115.912, 72.9577}	inf	19.	10 564.7	{996.165, 32 889.}	10 571.7	1.6057	newly	79.	7825.55	{599., 21 243.}	9806.85	1.53921	Reff	0.139655	0.800339	{0.19668, 2.1995}	473.107	ar	88 739.	203 214.	{172 029., 287 188.}	129.002
24	R0	{2.24089, 10.1789}	{1.94076, 8.36608}	{{0.743043, 2.74893}, {3., 12.1357}}	{49.3012, 36.6044}	inf	7.	5819.42	{1239.2, 6033.27}	1580.22	0.996441	newly	171.	4860.46	{875., 5975.}	2744.22	1.12872	Reff	0.025703	0.397888	{0.137455, 0.741601}	1448.02	ar	98 868.	192 693.	{174 481., 242 174.}	94.8994
25	R0	{1.57298, 10.2607}	{2.52464, 14.2308}	{{1.57894, 10.553}, {3., 17.6363}}	{61.1274, 41.4069}	inf	47.	6464.41	{1133.04, 13 048.7}	644.817	0.690864	newly	443.	3424.29	{526., 8310.}	677.867	0.668425	Reff	0.0819837	0.64361	{0.0456628, 1.93908}	699.343	ar	94 957.	184 154.	{161 812., 259 155.}	93.9345
26	R0	{1.2154, 8.2576}	{1.86558, 7.17096}	{{0.241153, 2.31141}, {3., 11.2042}}	{113.894, 51.7207}	inf	88.	2166.22	{541.867, 2689.58}	321.525	0.564049	newly	345.	2048.78	{272., 2987.}	504.417	0.595365	Reff	0.143952	0.225143	{0.016269, 0.455319}	101.029	ar	88 421.	157 699.	{151 156., 172 716.}	78.3501
27	R0	{2.22841, 18.7983}	{2.21023, 7.34371}	{{0.869004, 0.311362}, {3., 11.0583}}	{40.4363, 60.9342}	inf	67.	4966.76	{754.367, 2799.44}	106.137	0.310532	newly	1079.	3995.01	{596., 7260.}	286.985	0.376944	Reff	0.0322005	0.505488	{0.0181799, 0.983835}	1489.71	ar	98 756.	163 378.	{147 127., 190 773.}	65.436
28	R0	{1.27778, 5.36767}	{2.66367, 5.75504}	{{1.91796, 3.4022}, {3., 7.54211}}	{108.461, 34.9135}	inf	7.	6365.46	{226.622, 3993.76}	18 621.9	1.92874	newly	32.	5220.71	{247., 9537.}	16 214.7	1.91598	Reff	0.130065	0.463708	{0.249091, 0.711925}	256.52	ar	89 843.	177 665.	{164 831., 192 577.}	97.751
29	R0	{2.38042, 15.8901}	{2.2316, 4.55632}	{{1.06463, 1.0814}, {3., 11.3287}}	{34.8829, 72.2601}	inf	37.	7322.52	{2495.54, 6459.63}	221.65	0.21663	newly	849.	4941.31	{1644., 7302.}	484.267	0.611341	Reff	0.0237804	0.28467	{0.0560695, 0.401271}	1099.28	ar	99 112.	191 783.	{171 512., 229 590.}	93.5012
30	R0	{2.53188, 12.7607}	{2.28265, 5.90655}	{{1.74149, 1.62039}, {3., 8.52323}}	{25.7443, 53.713}	inf	11.	2405.77	{1024.04, 3130.59}	146.867	0.304406	newly	428.	7254.79	{631., 32 737.}	1598.18	0.716662	Reff	0.0182295	0.681788	{0.20229, 1.74147}	3640.02	ar	99 317.	175 704.	{155 744., 237 063.}	76.912
31	R0	{1.24482, 10.5471}	{2.51468, 4.68447}	{{1.75314, 3.31087}, {3., 12.8684}}	{102.011, 61.3075}	inf	170.	6242.45	{536.128, 7168.86}	408.644	0.573	newly	689.	9867.03	{675., 19 035.}	1343.53	0.858998	Reff	0.142009	0.713429	{0.318231, 1.74676}	410.819	ar	89 097.	197 700.	{174 273., 254 749.}	121.893
32	R0	{2.30446, 17.9607}	{2.50313, 11.1056}	{{1.96484, 3.64059}, {3., 16.3303}}	{22.7687, 38.1674}	inf	48.	7162.91	{994.526, 6142.91}	151.759	0.404269	newly	1094.	4881.08	{539., 10 531.}	368.314	0.519902	Reff	0.0266626	0.735982	{0.343344, 1.96482}	2660.35	ar	99 028.	179 291.	{156 552., 238 651.}	81.0512
33	R0	{2.31579, 11.8236}	{2.53345, 1.20697}	{{1.86561, 0.0333183}, {3., 6.35629}}	{26.0554, 89.7918}	inf	5.	327.688	{200.033, 517.263}	66.6896	0.361112	newly	197.	3004.87	{243., 1700.}	1425.31	0.426942	Reff	0.0234821	0.485169	{0.186762, 1.02194}	1968.17	ar	99 012.	166 999.	{159 683., 173 421.}	68.6651
34	R0	{1.6678, 10.6744}	{2.51478, 10.2344}	{{0.715736, 3.0675}, {3., 14.1219}}	{64.9873, 35.898}	inf	36.	4457.85	{608.524, 4502.03}	689.379	0.661699	newly	278.	2743.43	{463., 4212.}	891.234	0.733613	Reff	0.0712985	0.585114	{0.0812182, 1.20285}	723.864	ar	95 809.	171 710.	{155 014., 196 580.}	79.2207
35	R0	{1.65476, 7.07559}	{2.58087, 5.81092}	{{1.76709, 3.32116}, {3., 7.51799}}	{55.9664, 24.8764}	inf	5.	5712.37	{1840.09, 3655.09}	8181.97	1.66163	newly	50.	7922.7	{2095., 4006.}	15 746.7	1.74445	Reff	0.0705259	0.455008	{0.104315, 0.7748}	547.285	ar	95 746.	183 275.	{171 756., 176 855.}	91.4184
36	R0	{2.71807, 18.5959}	{2.66031, 12.0414}	{{2.02946, 1.2027}, {3., 18.6111}}	{15.6091, 35.9439}	inf	24.	12 037.	{4543.33, 13 276.}	225.557	0.420091	newly	1211.	10 605.1	{1885., 14 338.}	780.841	0.676835	Reff	0.0154386	0.721325	{0.146126, 2.02944}	4574.56	ar	99 540.	199 781.	{174 171., 255 412.}	100.704
37	R0	{2.6202, 12.7552}	{2.04593, 9.09483}	{{0.549102, 3.62574}, {3., 10.8416}}	{38.1981, 31.998}	inf	9.	10 327.2	{279.742, 33 823.}	1106.62	0.794324	newly	383.	7751.12	{159., 21 672.}	1944.32	0.924722	Reff	0.016088	0.405378	{0.0752922, 0.620214}	2421.22	ar	99 426.	185 487.	{167 383., 235 376.}	86.5577
38	R0	{1.47672, 9.81651}	{2.25232, 9.89659}	{{0.81826, 3.47077}, {3., 17.2397}}	{71.2619, 46.6963}	inf	40.	11 646.	{608.166, 38 182.4}	1953.2	0.955483	newly	323.	8439.86	{468., 23 395.}	2518.01	1.00213	Reff	0.0934468	0.577804	{0.183926, 1.28528}	525.368	ar	93 815.	176 181.	{147 180., 262 285.}	87.7962
39	R0	{1.90004, 12.3313}	{2.67042, 3.75792}	{{1.97005, 1.50903}, {3., 6.27783}}	{40.5453, 69.5253}	inf	30.	4529.87	{83.3721, 8048.68}	369.33	0.627901	newly	464.	7890.68	{372., 16 434.}	1617.39	0.919023	Reff	0.0517191	0.677153	{0.196016, 1.58002}	1209.29	ar	97 413.	187 341.	{164 151., 242 757.}	92.3167
40	R0	{2.64218, 18.5237}	{2.41593, 11.6953}	{{1.11723, 3.4363}, {3., 16.637}}	{25.3558, 36.8632}	inf	25.	6070.6	{4446.29, 7641.75}	124.016	0.280474	newly	874.	4150.59	{1502., 8832.}	379.168	0.52096	Reff	0.0162494	0.513025	{0.0330287, 1.08092}	3058.71	ar	99 474.	187 229.	{171 907., 241 185.}	88.2193
41	R0	{1.6945, 7.17996}	{2.44651, 3.49621}	{{1.8469, 0.648609}, {3., 5.81868}}	{44.3797, 51.306}	inf	4.	2913.74	{489.94, 6247.01}	2728.87	1.11246	newly	74.	5376.4	{464., 6672.}	7165.41	1.38851	Reff	0.0626626	0.568396	{0.0342455, 0.923977}	820.087	ar	96 332.	178 838.	{163 767., 205 881.}	85.6475
42	R0	{1.84559, 18.0074}	{2.74205, 13.4717}	{{2.28348, 7.88076}, {3., 18.5363}}	{48.5733, 27.6021}	inf	180.	3080.49	{289.703, 2317.96}	103.271	0.569005	newly	1374.	2265.38	{194., 1482.}	165.464	0.492882	Reff	0.0586713	0.580312	{0.222216, 0.985189}	889.552	ar	97 358.	191 173.	{178 493., 195 349.}	96.3612
43	R0	{2.24965, 17.0361}	{2.62249, 12.3289}	{{1.95136, 3.63925}, {3., 19.1125}}	{26.12, 34.8936}	inf	59.	7868.43	{1369.11, 12 329.5}	173.421	0.456523	newly	1018.	5380.42	{671., 9757.}	443.138	0.619257	Reff	0.0319	0.806938	{0.352371, 1.95134}	2429.58	ar	98 770.	182 863.	{164 085., 239 941.}	85.14
44	R0	{2.54324, 15.7967}	{2.59185, 3.88941}	{{1.92592, 2.80579}, {3., 4.30437}}	{20.3587, 75.8368}	inf	13.	2766.86	{786.272, 3683.39}	95.5832	0.235191	newly	626.	4262.61	{966., 10 125.}	592.788	0.652294	Reff	0.0177772	0.635139	{0.215792, 1.59493}	3474.32	ar	99 354.	194 673.	{178 596., 239 610.}	95.939
45	R0	{2.54943, 14.8032}	{2.20271, 7.30574}	{{0.717827, 1.93304}, {3., 16.2355}}	{36.5751, 55.7336}	inf	8.	6432.11	{306.415, 7225.19}	406.421	0.569221	newly	536.	3859.44	{380., 6185.}	633.739	0.665045	Reff	0.0170047	0.347914	{0.0522358, 0.736121}	1946.86	ar	99 380.	208 716.	{187 679., 251 806.}	110.019
46	R0	{2.37755, 12.6461}	{2.55495, 3.01887}	{{1.99241, 1.31134}, {3., 4.57246}}	{22.6257, 76.1281}	inf	9.	5301.7	{504.247, 3514.28}	516.442	0.525375	newly	377.	12 268.	{544., 36 248.}	3155.99	0.939767	Reff	0.0224678	0.633653	{0.17186, 1.99239}	2720.38	ar	99 096.	185 790.	{163 121., 256 971.}	87.485
47	R0	{1.69279, 11.6636}	{2.53425, 14.0963}	{{1.7464, 10.6401}, {3., 17.7459}}	{49.8433, 27.877}	inf	61.	10 083.2	{250.49, 19 374.4}	785.409	0.821017	newly	550.	4751.43	{96., 9338.}	791.962	0.817603	Reff	0.0676608	0.604531	{0.212675, 1.89229}	794.725	ar	96 214.	187 303.	{159 700., 275 222.}	94.6731
48	R0	{1.82402, 10.0038}	{2.25912, 7.39387}	{{1.20637, 2.2043}, {3., 9.5358}}	{48.4404, 26.0894}	inf	27.	7074.73	{4314.13, 4314.13}	1336.75	0.987471	newly	248.	5667.34	{2254., 6751.}	2185.22	1.14196	Reff	0.054447	0.322565	{0.0203065, 0.999542}	551.045	ar	97 076.	185 099.	{172 493., 202 273.}	90.6742
49	R0	{1.6424, 11.1667}	{2.62234, 4.09152}	{{0.628155, 2.37771}, {3., 4.72554}}	{75.9206, 66.4364}	inf	29.	2487.5	{2086.93, 2303.71}	270.859	0.517116	newly	321.	2655.05	{1679., 3399.}	727.118	0.84698	Reff	0.0752383	0.311763	{0.0922424, 0.580734}	318.002	ar	95 546.	180 832.	{175 136., 193 463.}	89.2619
50	R0	{2.05638, 14.3048}	{2.29781, 12.8284}	{{1.09509, 7.0816}, {3., 18.4888}}	{42.2848, 37.8525}	inf	38.	6627.81	{556.01, 11 812.2}	338.104	0.496158	newly	636.	4520.49	{186., 6927.}	632.769	0.619474	Reff	0.0397498	0.518464	{0.122903, 1.00106}	1209.4	ar	98 194.	179 655.	{160 852., 222 075.}	82.9597
